# Bike Share Usage and the Built Environment: A Review

**DOI:** 10.3389/fpubh.2022.848169

**Published:** 2022-02-21

**Authors:** Yuanyuan Guo, Linchuan Yang, Yang Chen

**Affiliations:** ^1^Department of Urban and Rural Planning, Tianjin University, Tianjin, China; ^2^Department of Urban and Rural Planning, School of Architecture, Southwest Jiaotong University, Chengdu, China; ^3^Department of Real Estate and Construction, Faculty of Architecture, The University of Hong Kong, Pokfulam, Hong Kong SAR, China

**Keywords:** micro-mobility, bikesharing, bike-sharing, physical environment, land use, urban design, living environment, urban environment

## Abstract

Bike share engages riders in physical activity, beneficial to health. In addition, it promotes green mobility and contributes to carbon neutrality. An understanding of the association between bike share usage and the built environment is essential for system operation/management and urban/transportation planning. Limited reviews of the existing literature exist now. Therefore, we review previous studies to decipher the complex relationship between bike share usage and the built environment. We focus on a few built environment dimensions (e.g., land use, transportation system, and urban design) and find that many attributes affect bike share usage differently across mobility cultures, between docked and dockless bike share, and between arrival and departure usage patterns. The effects of several attributes (e.g., proximity to a park or university and metro station density) on bike share usage also vary between weekdays and weekends and across trip purposes. The findings of this paper advance knowledge on the connection between bike share usage and the built environment.

## Introduction

Cycling is an economical, healthy, and green transport mode that contributes to carbon neutrality. Given these benefits, an increasing number of cities throughout the world are promoting cycling in ways such as by introducing the prevailing programs of bike share systems (1). Since its introduction in the 1960's, bike share has witnessed a worldwide prevalence and has proliferated in recent decades. The number of cities with bike share programs has increased from a sprinkling at the end of the 1990's to more than 800 in 2015, with more than 900,000 shared bikes equipped (2). Recently, dockless (floating) bike share has become prevalent, acting as a catalyst for globally promoting bike share development and cycling activities. In China, within only one year since the first operation of dockless bike share in 2016, the number of shared bikes has increased to over 23 million (3).

A burgeoning body of studies that examine bike share with various interests mirror the tremendous global growth of bike share programs (4). Related studies cover a range of topics, including its historical development, environment/economic/social benefits, basic features of usage, operation issues of rebalancing, optimization of station locations, promotion of the brand, theories of bike share adoption, determinants of usage, and policy/planning implications [e.g., (3, 5–14)]. Much research has explored the influencing factors of bike share usage under various contexts. Compared with the socio-demographic, natural environment, psychological, and attitudinal determinants, built environment features have received the most scholarly attention [e.g., (13–21)]. Research findings are diverse, but the consensus seems rare under different urban contexts and mobility cultures. Studies on how the built environment affects the usage of docked and dockless bike share differently are also insufficient (22). A literature review on the complex relationship between bike share usage and the built environment is needed to summarize the major research findings, identify research gaps, and point out future research directions. To the best of our knowledge, no such review has been conducted. We believe a comprehensive examination of the relevant studies should be of considerable importance for system operation/management and urban/transportation planning.

To this end, this paper provides a critical review of the existing literature on the connection between bike share usage and the built environment. International studies presented in English on exploring the association between bike share usage (e.g., docked and dockless patterns) and built environment are reviewed. The main focus of this paper is discussing the difference in the effects of the built environment on bike share usage in various conditions, such as mobility culture, docked vs. dockless bike share, trip purposes, arrival vs. departure usage pattern, and day of week.

The remainder of this paper is structured as follows. Section 2 presents an overview of previous studies and related built environment attributes. Section 3 presents the variance in the effects of the built environment on bike share usage. Section 4 recommends future research directions. The last section summarizes the major findings.

## Overview of Previous Studies and Related Built Environment Attributes

The built environment is the set of all physical parts of the environment. As Handy et al. (23) defined, the built environment is composed of three parts: *land use, transportation system*, and *urban design*. First, land use refers to the distribution of different types of land, as well as the location and density of various activities across the space. Land use directly affects access to the destination from the origin. Second, the transportation system typically includes the physical infrastructure (e.g., roads, sidewalks, bridges, and bike lanes) for transport support. By providing connections between different activities, the transportation system affects how easily an individual can reach his/her destination from an origin. Last, urban design refers to the appearance and arrangement of physical elements (e.g., the shape of the block and tree shadows), which affects the mode choice by influencing an individual's attractiveness judgment and safety perception (24). Furthermore, some researchers expand the dimensions of the built environment by the introduction of the urban form (e.g., population density and job density) (25–27).

**Figure 1:**
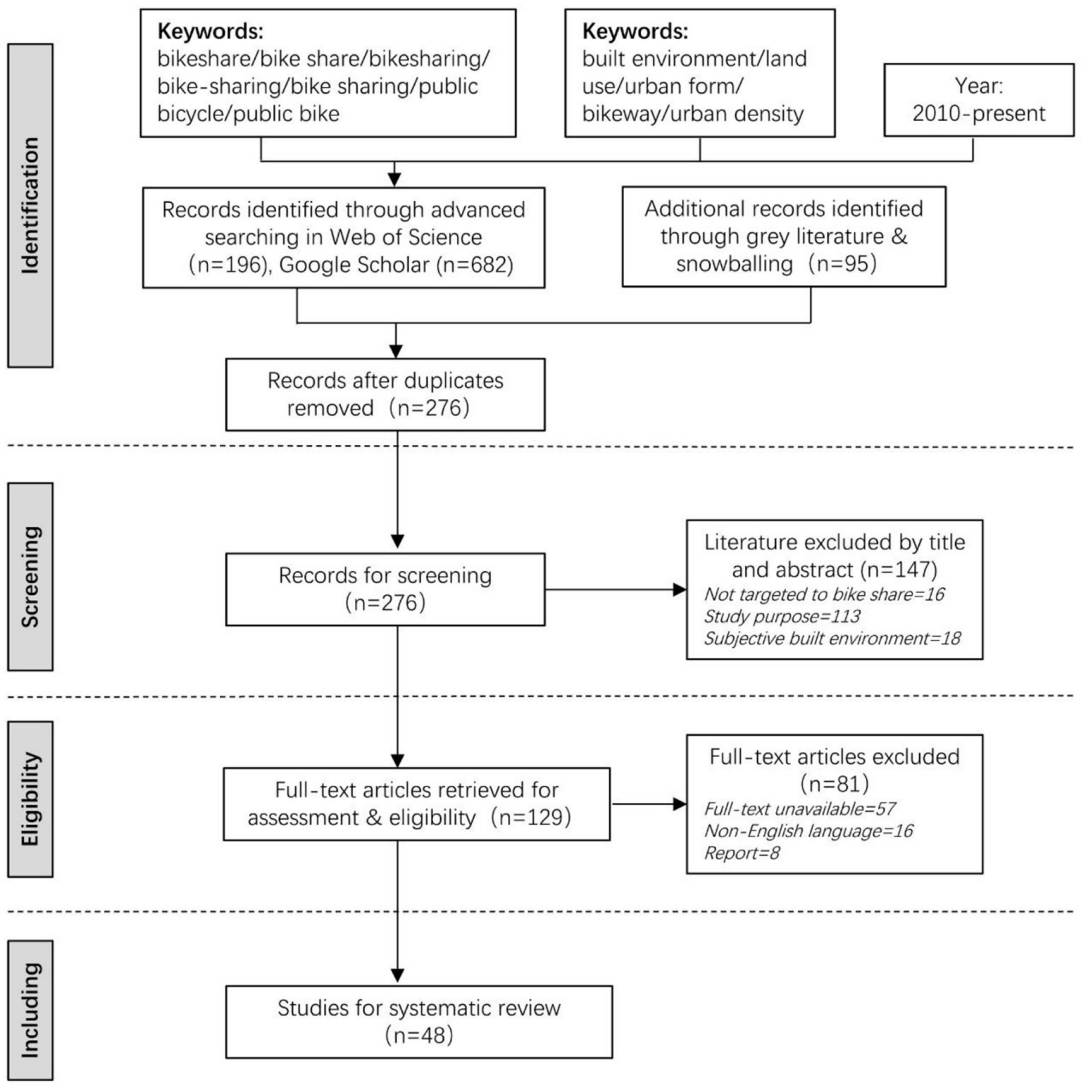
Flow diagram of the literature search and selection process.

In this study, we searched relevant papers through Web of Science and Google Scholar as well as gray literature (Figure 1). Relevant papers were also extracted by backward snowballing (28). We collected papers published since 2010 (when bike share studies merged), and keywords that are related to the built environment (or land use, urban form, urban density, bikeway) and bike share (or bicycle sharing, bikesharing, bike sharing, bike-sharing, public bike, public bicycle) were used to search articles. We only focused on the objectively measured built environment rather than the subjectively assessed built environment. Additionally, studies not correlated to bike share usage were excluded.

**Figure 2:**
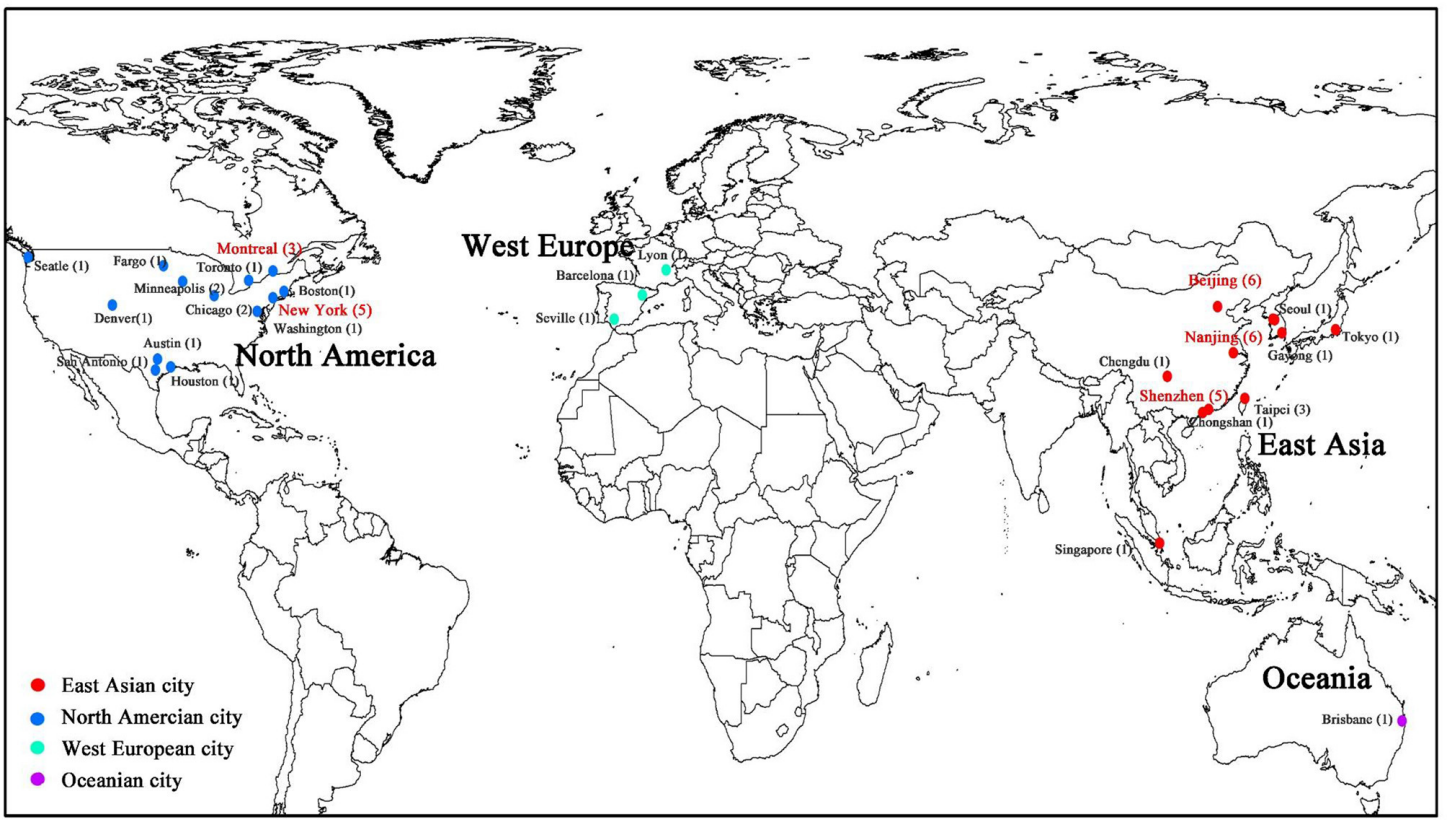
Studies on bike share usage and the built environment. The number of studies using the city as the study area is shown in parentheses. Popular study areas are marked red.

In total, 48 papers were finally retrieved: 19 were from North America, 24 from East Asia, two from West Europe, and one from Oceania (Figure 2). The other two papers made a comparative study worldwide. In terms of the operation model, 37 papers were pertaining to docked bike share, while ten were related to dockless bike share. The other paper compared the different effects of the built environment on two types of bike share.

The built environment elements often concerned in bike share usage studies are summarized and classified in Table 1.

**Table 1:** Categories and measurements of built environment features related to bike share usage.

**Category**	**Sub-division**	**Measurements**
Land use	Land use type	Percentage/areas of residential land use
		Percentage/areas of office land use
		Percentage/areas of industrial land use
		Percentage/areas of commercial land use
		Percentage/areas of green land use
	Mixture of land use	Entropy index of land use
	Activity-related sites/Points of Interest (POIs)	No. of shopping malls
		No. of/presence of universities/schools
		No. of/presence of parks
		No. of recreation sites
		No. of restaurants
		No. of retail stores
Transportation system	Urban road	Presence/length of bicycle lanes
		Length of main/major road
		Length of branch/minor road
		Length of highway/regional road
		Presence of a paved trail
		No. of street intersections
	Transit	No./presence of subway/rail stations
		Length of subway/rail
		No. of bus stops
		Length of bus lines
	Bike share facility	No. of bike share stations
		Capacity of docks
Urban design	Amenity	Street tree/shadow
		Street light/lamp
	Accessibility	Distance to city government
		Station distance to CBD
		Transfer distance to transit
Urban form	Density	Population/household density
		Employment/job density

In addition to Handy et al.'s model, another built environment assessment model is the so-called “3Ds,” “5Ds,” or “7Ds” models. The “3Ds” model was first proposed by Cervero and Kockelman (29). In this model, built environment attributes are categorized into three dimensions, namely *density, diversity*, and *design*. The “3Ds” model was later extended into the “5Ds” model by adding two dimensions of *distance to transit* and *destination accessibility*, and the latter was expanded into the “7Ds” model by incorporating *demand management* and *demographics* (30). The “Ds” models have been widely applied in travel behavior-built environment studies ever since.

## Variances in The Relationship Between Bike Share Usage and The Built Environment

Many variances exist in the relationship between bike share usage and the built environment. First, cities in West Europe, East Asia, and North America & Oceania are generally cycling-oriented, transit-oriented (or transit-dependent), and automobile-oriented, respectively (26, 31–33). Such a variance in the mobility culture results in a unique built environment related to cycling, thereby making a difference in affecting bike share usage. Second, recent studies found that docked and dockless bike share systems are generally different in user demand and travel characteristics (34). The urban built environment should determine docked and dockless bike share usage patterns differently. Third, bike share trips are often work- and school-related, followed by entertainment- and recreation-related. Bike share is also frequently used to connect transit, providing an efficient solution to the first/last mile issue (35). In general, these diverse bike share trip purposes are associated with distinct urban built environment features. Fourth, bike share usage is typically identified with the arrival (drop off) and departure (pick up) patterns. Therefore, the built environment around origins and destinations is important to determine bike share usage (36). Last, bike share usage varies between weekdays and weekends. Due to the daily variance in demand for bike share, the built environment affects bike share usage differently between weekdays and weekends (37).

Five perspectives of exploring the variance on the association between bike share usage and the built environment can be identified and are discussed: mobility culture, bike share type (i.e., docked vs. dockless type), trip propose, usage pattern (i.e., arrival vs. departure), and day of week.

### Variance of The Built Environment Effects Across Mobility Culture

#### Mobility Culture of Cycling in West Europe, East Asia, and North America and Oceania

In West European countries, cycling is viewed as a norm rather than an exception. The well-designed cycling environment and facilities make the bicycle a major transport mode (38). For instance, cycling accounts for 10%−37% of commute trips in large bike-friendly cities in the Netherlands, Denmark, and Germany (39). Cycling trips are common across all demographics. People often use bikes to reach the workplace, school, daycare, grocery stores, and events. Cyclists tend to enjoy a high level of protection in traffic because of the bike-friendly environment (40). Moreover, urban planning and policies encourage cycling, such as providing space for bicycle parking (e.g., bike share docks) instead of only for motor vehicles. Bike lanes are usually clear and well maintained (41).

As a result of a dramatic transformation brought by rapid urbanization, dense East Asian cities are often characterized by mixed land use and diverse communities (e.g., residential community and urban village). Such a characteristic theoretically encourages cycling behavior because of the short travel distance. However, many metropolitan areas, such as Tokyo, Beijing, and Seoul, have a high mode share of transit and automobiles but a small proportion of cycling (35). In particular, China was once well-known as the “Kingdom of Bicycles” in the 1980's but has experienced the rapid growth of private automobiles and the unprecedented expansion of transit since the 2000's. Cycling facilities and environments are often poorly maintained. Therefore, the mode share of bicycles has dropped sharply. However, the popularity of bike share (public bicycle) has increased since circa 2008, facilitating the investment of cycling infrastructure and promoting cycling behavior.

In North America & Oceania, the city is often low-density and low-mixture, making the car a common choice of transport mode. Furthermore, suburbanization encourages long-distance motorized trips. As a result, cities are more likely to be automobile-oriented and unsuitable for non-motorized travel (e.g., walking and cycling). Bike culture in these countries seems non-mainstream. In many cities, less than 3% of residents cycle to work, indicating that the bicycle has a low mode share (31). The bicycle is popular among groups who pursue healthier and more sustainable lifestyles, particularly for recreational and sporting trips rather than utilitarian trips (e.g., commute and going to school). However, recently popular bike share programs have offered citizens a fashionable notion of cycling and encouraged an increase in cyclists, spurring a “cycling renaissance” since the 2010's (42).

#### Different Effects of the Built Environment on Bike Share Across Mobility Culture

Many built environment features have an inconsistent effect on bike share usage among different mobility cultures. The differences in the effects of the built environment on bike share usage are as follows:

(1) The (positive) effects of commercial and office land use in East Asia differ from those in North America (20, 43). Bike share is frequently used around the workplace in East Asian cities but not in North American cities (44).
(2) Restaurants near bike stations in West Europe and North America attract a large number of bike share trips (15, 37, 45). However, this case does not apply to East Asia.
(3) People in West European cities often cycle to retail stores, indicating that the bicycle is a persuasive mode of transport in daily life, while in North America, cycling is not commonly adopted for this purpose (43, 45).
(4) In East Asia (e.g., China), docked bike share systems in many cities have been widely distributed in urban areas. Thus, adding bike stations in such areas may have a marginal effect on the average ridership, so it is not always advocated (13). By contrast, the number of bike stations in North American cities is small. Therefore, in such contexts, an increase in the density of bike stations to promote the network effect is often called for (17, 37, 46).
(5) Owing to the difference in urban density between East Asia, West Europe, and North America, population/employment density in the catchment areas of bike stations affects the demand for bike share usage differently. In West Europe and North America, city centers, where population and jobs are concentrated, have a high demand for bike share usage (46). However, in East Asian cities (e.g., Seoul, Beijing, Taipei, and Shanghai), dense areas may have crowdedness and congestion problems, thereby decreasing the willingness to use bike share because of the increased risk of crashes with pedestrians (20, 34, 47).

### Variance of the Built Environment Effects Between Docked and Dockless Bike Share Patterns

Docked bike share users need to rent bikes from docking stations near the origin and then return them to the stations near the destination. By contrast, dockless shared bikes can be used in nearly all public spaces. Thus, their distinct operations result in different travel patterns and characteristics and varying associations of their usage with the built environment (34). For docked bike share systems, the built environment around designed bike stations is relatively targeted. However, the urban built environment among the areas for dockless shared bike pick-up or drop-off should have a wide variety and uncertainty.

Dockless bike share and docked bike share are the typical modes of cycling transport. As many studies indicated, cycling facilities (e.g., bikeway) and mixed land use are often associated with cycling behavior. This observation evidently applies to two patterns of bike share, as presented in Supplementary Table A1. However, several built environment attributes influence the usage of docked and dockless bike share differently. These differences are summarized as follows:

(1) Dockless shared bikes are more distributed around residential and industrial areas than docked shared bikes. The freedom of bicycle parking makes residents and factory workers use bikes conveniently, thereby generating a high demand for bike share usage in residential and industrial areas. Owing to the heavy investment in installing bike stations, distributing docked bikes as widely as dockless bikes in residential areas is difficult, if not impossible. In most cases of urban China, a residential community is at most equipped with one bike station, whereas dockless shared bikes can be discretionarily dropped off by users or designedly allocated by operators at several entrances/exits (48). Dockless bike share, which enables a quick connection for the factory-residence short commute, is also popular among workers who live outside factories. It is cheap and convenient (14). However, docked bike share is less frequently used because of the high deposit and the complicated process of membership registration (e.g., 300 RMB in Shenzhen).
(2) Compared with docked bike share, dockless bike share is more attractive in connecting public buses. As Supplementary Table A1 shows, most dockless bike share research (except for that related to the metro) presents the effect of the bus service on promoting bike share usage, whereas only a few studies on docked bike share indicate similar results. A possible reason for such a variance is that bike stations are designedly installed around metro stations but rarely put around bus stops. Another possible reason is that the trip distance of dockless bike share is generally shorter than that of docked bike share (34).
(3) Docked bike share stations are often installed in city center areas but seldom distributed in suburban and exurban areas, possibly due to the government's concern of low ridership. Therefore, the further away from the CBD, the lower the bike share usage (49). However, the negative association between the distance to the CBD and bike share usage is not applicable to dockless bike share. For many Chinese cities, where dockless bike share is very popular, suburban areas have a high volume of dockless bike share usage (even higher than city centers in some cases). This phenomenon is evidenced by two other built environment attributes, namely population density and job density, which reportedly have no statistically significant effects (28, 34, 50, 51). Some research explained that bicycle parking space for dockless bike share is limited in city centers (52). Another possible explanation is that city center areas have good walking accessibility, resulting in minimum demand for cycling (14). A recent study has explored the nonlinear effect of various density indicators on dockless bike share usage (53). It concludes that cycling trips consistently increase when employment density and population density reaches 12,000 jobs/km^2^ and 20,000 persons/km^2^, respectively. After exceeding the thresholds, the effects of employment density and population density are minor.

### Variance of the Built Environment Effects by Trip Purpose

#### Commute and Leisure Usage

Job/employment density is one of the determinants of the demand for bike share commute (15, 20). In terms of land use types, residential land and commercial land usually contribute to a large volume of bike share commute trips (54). In addition, school commute by bike share is well adopted by university students (17). This observation indicates that the closer proximity of a university to bike stations helps to increase ridership. Another factor affecting bike share usage for commute is trip distance. Either docked or dockless bike share users prefer a short trip (34). A long trip may make commuters hesitate to choose bike share as their major commute mode. Instead, commuters are more likely to shift toward transit or automobiles (17, 55). In light of this, bike share users usually have a strong preference for a shorter trip distance (56).

Given that the leisure purpose of bike share usage is associated with recreational activities, the installation of bike stations needs consideration of proximity to recreation sites. Chen and Ye (53) indicated that an increasing number of leisure services in the traffic analysis zone (TAZ) generate continuous growth in dockless bike share usage when the number reaches 37. Faghih-Imani et al. (45) indicated that in Spanish cities, recreation POIs were one of the important contributors to high bike share ridership. These sites include parks, cinemas, lakes/rivers, places of historical interest, tourist areas, and other recreation areas (37, 57). Of these sites, the park is the place that attracts most leisure trips by bike share. Loop journeys (starting and ending at the same station) are particularly popular in parks (54). The presence of parks also helps promote dockless bike share usage because parks are attractive destinations for recreational activities (51). Furthermore, an empirical study on the Vélo'v program shows that recreational activities after work (e.g., going to restaurant/cinema/other recreational places) mainly happen in city centers (37). This finding indicates that city centers with recreation facilities tend to attract more leisure trips by bike share, consistent with the research of Mateo-Babiano et al. (54). In addition to the presence and location of recreational sites, the proximity of recreational sites to bike stations is a crucial factor that fundamentally affects the demand for leisure trips by bike share. Wang et al. (16) found that the shorter the distance from bike stations to the lake, river, waterfront, and parks, the larger the ridership of docked bike share.

#### Feeder Mode of Connecting Transit

Bike share contributes to achieving the goal of seamless integration with transit, thereby providing a promising solution to the first- and last-mile problems. Among the factors affecting the feeder mode choice of the metro, the built environment around the metro station/home/workplace plays an important role.

Supplementary Table A1 indicates that the effects of land use on the bike share–metro integration are not always consistent among different urban contexts. Ni and Chen (48) found that residential and office land uses are associated with a high demand for dockless bike share–metro integration. Guo and He (14) identified a positive association between the integrated usage and industrial land use Guo et al. (21) determined a positive association between land-use mixture and integrated use. As for docked bike share–metro integration, Ji et al. (19) pointed out that commercial land use is contributory. Zhao and Li (44) found a negative effect of the shopping mall factor. Furthermore, public parks around metro stations increase the probability of connecting metro stations by (docked and dockless) bike share because cyclists may travel through the park to avoid traffic, injuries, and traffic lights (14, 44).

Regarding transportation facilities, the role of the bikeway in determining integrated usage is not as important as the common usage. Several studies pointed out that dedicated bicycle lanes fail to encourage integrated usage because they are always illegally occupied by cars (14, 44). The distribution of transit also differently affects the bike share–metro integrated usage from the common usage. As one of the feeder modes of metro transit, public buses can substitute bike share on connecting the metro station (56). Therefore, the high-quality bus service (i.e., bus stops/lines) in metro catchment areas results in increasing competition between bike share and public buses (14, 19, 48, 58, 59), thereby reducing the bike share–metro integration. Notably, the transfer distance of connecting the metro station is the key determinant of bike share–metro integration. A moderate transfer distance between 0.8 and 1.5 km is suggested to be appropriate for frequent bike share–metro integrated usage (44). Some Chinese empirical evidence indicated that dense distribution of metro systems reduced the potential of integrated usage (14, 19, 35). The reason for this result is that areas with many metro stations have a short transfer distance, thereby promoting the likelihood of walking, rather than bike share, as a feeder mode. By contrast, for common usage of bike share, areas with many bus stops or metro stations have been often observed with a high level of bike share usage (28, 50, 60–63).

### Variance of the Built Environment Effects Between Arrival and Departure Patterns

#### General Arrival and Departure Usage

Bike share users need to rent bikes from docking stations near their place of origin (departure) and return bicycles to bike stations around their destination (arrival) (64). As such, the average daily statistics are calculated on the basis of borrowing and returning records. By influencing the accessibility and convenience of renting a bike share, the built environment may have different associations with borrowing and returning behavior, particularly for docked bike share [e.g., (15, 16, 34, 43, 45, 63, 65)]. In a nutshell, built environment attributes related to land use mostly contribute to the variance between arrival and departure usage patterns. However, transportation, urban form, and urban density-related built environment factors have few variances in their effects on the two types of bike share usage.

Among the selected literature, studies by Sun et al. (43) and Liu and Lin (20) present different effects of land-use built environment on the arrival/departure bike share usage. Office and green land use around bike stations are negatively correlated to bike share pick-up, whereas fewer returns of bike share occur at commercial areas. This finding suggests that bike share users are less likely to set out around the places of business districts and parks and choose commercial sites as their cycling destinations (43). Liu and Lin (20) found that the areas with mixed land use and many recreation sites often have high arrival usage instead of departure usage. However, bike stations with many surrounding restaurants (e.g., coffee shops and bars) have a high volume of arrival and departure usage (15, 18, 45, 62, 63).

Significant variables related to transportation facilities in the departure (trip origin) model and their influencing direction are almost the same as those in the arrival (trip destination) model. The only striking difference is their magnitude (11, 15). Among the most selected attributes, facilities related to urban roads have shown significant effects on arrival/departure usage. For example, bikeways often contribute to a high willingness to cycle by offering bike share users a safe means of travel (17). Branch/minor roads in the catchment area of bike station are positively associated with the arrival/departure usage of bike share, whereas the highway, railway, and major/main road are negatively associated (11, 12, 15, 17). The presence of a subway station near the bike station may raise the trip frequency for departure and arrival [e.g., (37, 65)], indicating the potentials for connecting the transit. Dock capacity is also a key factor affecting departure and arrival use simultaneously, which has been evidenced by a series of empirical studies [e.g., (17, 49, 63)].

Regarding the urban design, street trees and lamps, which may be related to a safe and comfortable biking environment, increase the departure usage rather than arrival usage (20). This result can be interpreted as being indicative of users' preference for starting a trip in a good biking environment. Urban density, in terms of population and employment, affects arrival and departure usage positively [e.g., (15, 18, 43, 45, 66)]. Bike stations in dense areas with many people and job opportunities generally have higher arrival and departure ridership than those in other areas because of the generation of a larger number of trip demands.

#### Arrival and Departure Usage Related to Time of Day

The operation experience of cities indicated that temporal dynamics of arrival/departure bike share usage are always observed with variations throughout the day (14, 54). Such temporal variance in the usage is partly attributed to some unique determinants of the built environment, potentially strongly affecting the trip attraction and generation at a specific time of a day (17, 37). Faghih-Imani and his colleagues have conducted several studies on exploring the different effects of built environment attributes (i.e., POIs of restaurant and university, and population and employment density) on the bike share arrival/departure usage in the morning, afternoon, and evening (11, 12, 15, 18, 45).

Through establishing interaction items of built environment attributes and time period, they found that restaurants in the vicinity of a station are irrelevant to bike share usage in the morning but positively associated with that in the evening, possibly because restaurant shops are usually not in opening hours in the early morning (15). Bike share stations near or within the university also experience a temporal variance. In the morning, the arrival rate is high, but the departure rate is low. Totally opposite results were observed during the evening time. A possible explanation is that a number of university students often ride a bike to school from their outside residence (11, 15). Their outcomes were also evidenced by Tran et al. (37). Areas with denser populations are also likely to have less arrival but more departure usage in the morning for bike share members. However, the effect of population density is not clear in terms of afternoon and evening (11, 12, 18). A positive effect of job density on the arrival usage in the morning and departure usage in the afternoon is also observed from several case studies (12, 15, 18).

### Variance of the Built Environment Effects by Day of Week

Usage of bike share on weekdays and weekends has been identified with striking differences. On weekdays, home-to-work commuters are the majority of bike share users, where on weekends, bike share is used for diverse purposes (e.g., shopping, sports, visiting). The turnover of (docked and dockless) bike share on weekdays is significantly larger than that on weekends (15, 17, 51). Such a difference in bike share usage is not only derived from the calendar attribute itself but also from built environment features, which take effects differently by influencing trip generation (67, 68). In particular, some built environment elements have been demonstrated to only work on weekdays or weekends (12, 13, 17, 18, 47, 69).

Among several selected land use types, the residential land use displays a similar effect on bike share usage on weekdays and weekends (47). During weekdays, users tend to pick up or drop off bikes at the station near their homes with the purpose of commute. However, Noland et al. (69) pointed out the association between residential land use and bike share usage is positive on weekends but insignificant on weekdays. Leisure/shopping trips starting from and ending at home are more popular on weekends than on weekdays (13). Office land use also shows an opposite effect between weekdays and weekends. It is negatively associated with bike share usage on weekends but positively on weekdays because of an increase in commute activities on weekdays (47). The contributor of mixed land use is more likely to promote bike share usage on weekends than weekdays, possibly because of diverse purposes of outside trips on weekends, such as shopping, leisure, sport, and visiting.

Park and university also show some variance of their effects on the usage between weekdays and weekends. Stations near a park have an increase in bike share traffic volume on weekends. Notably, these stations enjoy approximately twice the amount of traffic on weekends than on weekdays because of the growth in leisure activities at parks (12, 13, 18). By contrast, the presence of a university near the station is more likely to improve bike share usage significantly on weekdays than on weekends (17). A possible reason for this result is that university students living outside but close to the campus tend to adopt bike share to access the campus for class on weekdays.

Supplementary Table A1 also indicates a few variances between weekdays and weekends in terms of the effects of the transportation-related (e.g., bikeway, urban road, and bike share facility) and design-related (e.g., street trees, lamps, and distance to CBD) built environment on bike share usage. Given that the integration usage with transit is often aimed for commute, the presence of the metro/subway near the bike station is significantly associated with a high volume of bike share ridership on weekdays (17). However, improving the bike share ridership becomes less important on weekends due to fewer trips by the metro (47).

Population density is positively correlated with bike share trip generation, and few variances between these two periods of week occur on affecting bike share usage. However, the effect of job density on bike share usage varies between weekdays and weekends. Job-dense areas can attract a large number of bike share usage (arrival and departure patterns) on weekdays because of frequent commute trips. By contrast, scarce commute activities occur in these areas on weekends, resulting in the minimal potential for bike share trips (17, 69).

## Recommendations for Future Research

This review of the literature discussed the built environment factors that are most associated with the usage of docked and dockless bike share. However, a more comprehensive approach is still needed to obtain better insights into the effects of the urban built environment on bike share usage patterns, thereby informing policymakers to appropriately encourage shared mobility. Further studies are needed to broaden the research in this field.

This study has shown that the effects of certain built environment factors on bike share usage were sometimes inconsistent among the selected empirical studies. In addition to the possible reason for urban context variances, this inconsistency is attributed to the complex nonlinear relationships between built environment and travel behavior (70, 71). In particular, built environment factors related to density, such as density of POIs, urban road, intersection, population, and employment, are often evidenced with nonlinear effects on travel behavior. However, a handful of studies have paid attention to the nonlinear association between the built environment and bike share usage (43, 53, 60, 61). More studies are expected to detect the nonlinear relationship by applying the newly proposed methodology of machine learning, such as the gradient boosting decision tree model (71). Furthermore, the inconsistent findings may be due to the poor quality of methodologies. Cross-sectional studies, which can detect associations but cannot infer causality, now dominate this research field. We suggest collecting longitudinal data and conducting more sophisticated studies in the future to reach more persuasive conclusions and perhaps reconcile the discrepancy in research outcomes. In doing so, safer policy/practical implications can be drawn.

Given an increasing integrated usage of bike share and transit, more studies continue to be needed on how the urban built environment shapes the bike share-metro and bike share-bus integrations. Although several relevant studies have been conducted (19, 44, 58, 59, 72), these studies are lacking in identifying the different effects of the built environment on several types of bike share-metro integration, including the access to transit (from home or workplace) and egress from transit stations (to home or workplace). Additionally, the built environment around home/workplace, along the access/egress trip, and around the metro station also have different effects on choosing bike share as a feeder mode. Without considering the integrated types, the real effect of the built environment may be concealed, thereby misleading policymakers and decision-makers and resulting in inefficiency.

Rather than only supplementing the metro, bike share also competes with metro transit in certain areas and time periods. Melbourne's experience suggested that bike share was potentially substituting for transit rather than connecting to it (9). This phenomenon has also been demonstrated in London, where 35% of bike share users reported a substitution for subway usage and a reduction of overcrowding transit at peak hours (73). Therefore, the substitution effect accompanies the integration effect simultaneously, and it cannot be ignored (55). Then, how does the built environment determine the direction of bike share usage in terms of the relationship with transit? Is it inclined toward the integration with metro transit or modal shift from metro transit? Further explorations on the influences derived from the built environment should be the subject of future research.

We still do not know how the perceived built environment influences bike share usage. Most empirical studies measure the built environment elements by online map (e.g., OpenStreetMap), census data, and GIS data (74). These approaches are objective ways to depict reality. However, travel behavior is not only affected by the built environment but also by an individual's perception, which has more direct effects (75). Therefore, the subjective measurement of the built environment by considering individual perception is of vital importance to enrich studies on the association between bike share usage and the built environment (76, 77). Some researchers have also revealed a mismatch between objectively and subjectively measured built environments. Hence, the effects of the two types of the built environment on travel behavior should be different (76). One study has been conducted recently to identify how the objective and perceived built environments affect dockless bike share usage differently. However, more concentrations on this stream of work are still needed.

Finally, comparative studies among cities within a county or across different cycling cultures remain scarce. Characteristics of the built environment in North “America”. Europe, East Asia, and Oceania are significantly distinct (78). Such variance may lead to different results in terms of the effect of the built environment on bike share usage, and reaching consistency is difficult. Additionally, under the influence of the risk of exposure to worldwide COVID-19, a safe built environment could be fundamental for encouraging bike share usage for cyclists to protect from the COVID-19 while keeping healthy via physical activities. In this sense, comparative studies worldwide in this streamline are essential.

## Conclusions

From the perspective of the urban environment and society, bike share offers a number of advantages over other transport modes. Therefore, the usage of bike share can be encouraged for many reasons, and academic researchers and local governments have paid considerable attention to relevant topics in recent years (79–84). For encouraging cycling and promoting bike share programs, we need a better understanding of built environment content to derive more insights into what factors of the built environment could play an important role in influencing bike share usage.

Studies on the effects of the built environment on bike share usage have been recently conducted worldwide. However, current empirical findings are dispersed, which inspires us to do this work. Our purpose is to identify the built environment determinants of bike share usage pertaining to land use, transportation system, and urban design. In terms of the effects of the built environment on bike share usage, several variances among worldwide mobility cultures, between docked and dockless bike share patterns, among split travel purposes, between arrival and departure usage, and between weekday and weekend time have been systematically reviewed. We think that our findings could not only enhance the understanding of bike share usage but also provide a useful reference for urban transport planning. We provide a summary of the major findings as follows.

(1) Built environment and mobility culture vary among East Asia, North America, Oceania, and West Europe, thereby resulting in variances in the characteristics of bike share usage. Several built environment features (e.g., office/commercial land use, distributions of parks, restaurant, and retail POIs, bike station network, and urban density) affect bike share usage differently under the distinct mobility cultures.
(2) The effect of the built environment on bike share usage differs between docked and dockless patterns of bike share in several aspects. First, residential/industrial areas are observed with more dockless bike share usage than docked bike share usage. Second, in addition to the strong integration with transit, dockless bike share has greater potential than does docked bike share for bike share–bus integration. Third, dockless bike share also has a broader distribution than docked type across urban space, such as suburban areas with relatively low density of population/job.
(3) Trip purposes by bike share are typically correlated with unique built environment attributes. Residential/office/commercial land use, presence of school/university, distance traveled, and job density are the major determinants affecting the bike share demand of commute, while leisure trips by bike share are correlated to the distribution of recreation POIs (e.g., park). Moreover, the function of bike share on connecting with transit is mostly affected by how the metro station is distributed, which fundamentally determines the distance to transit. Notably, bike share–metro integration is also significantly affected by bus service in the metro catchment areas.
(4) Built environment attributes related to land use (e.g., office and green land use) mostly contribute to the variance between arrival and departure usage patterns, while transportation-, urban form- and urban density-related built environment factors contribute few variances. The effect of several selected built environment attributes, such as restaurant POIs, university/school, and population/job density, on the arrival/departure bike share usage also depends on time of day (i.e., morning, afternoon, and evening).
(5) Considerable differences have been observed in bike share usage between weekdays and weekends, possibly due to the different trip attractions/generations derived from specific built environment attributes. Built environment attributes related to residential/office land use, park, university, presence of the metro/subway (or metro density), and job density present many variances of the effects between weekdays and weekends.

## Data Availability Statement

The raw data supporting the conclusions of this article will be made available by the authors, without undue reservation.

## Author Contributions

YG: conceptualization, formal analysis, methodology, and writing – original draft. LY: conceptualization, supervision, funding acquisition, and writing - review & editing. YC: conceptualization, formal analysis, methodology, validation, and writing – original draft. All authors contributed to the article and approved the submitted version.

## Funding

This study was supported by grants from the Science and Technology Department of Sichuan Province, China (No. 22RKX0638) and the National Natural Science Foundation of China (No. U20A20330).

## Conflict of Interest

The authors declare that the research was conducted in the absence of any commercial or financial relationships that could be construed as a potential conflict of interest.

## Publisher's Note

All claims expressed in this article are solely those of the authors and do not necessarily represent those of their affiliated organizations, or those of the publisher, the editors and the reviewers. Any product that may be evaluated in this article, or claim that may be made by its manufacturer, is not guaranteed or endorsed by the publisher.
