# Supplementary material for: Bike Share Usage and the Built Environment: A Review
Source: Front Public Health. 2022 Feb 21;10:848169. doi: 10.3389/fpubh.2022.848169 (PMC8898941; doi:10.3389/fpubh.2022.848169)
Supplement: Supplementary file 1 [file Table_1.docx]

**Supplementary Table A1 Summary of selected empirical studies**

| Type | Category | Research | | Land use | | | | | | | | | | | | Transportation facility | | | | | | | | | | | | | Urban design | | | | Urban  Form | |
| --- | --- | --- | --- | --- | --- | --- | --- | --- | --- | --- | --- | --- | --- | --- | --- | --- | --- | --- | --- | --- | --- | --- | --- | --- | --- | --- | --- | --- | --- | --- | --- | --- | --- | --- |
|  |  |  | | Residential land use | Office/business land use | Industrial land use | Commercial land use | Green land use | Entropy index of land use | No. of shopping malls | Presence of college/school | No. of recreation sites | No. of /presence of park | No. of restaurants | No. of retailing stores | Presence /length of bicycle lanes | Length of main/major road | Length of branch/minor road | Length of highway/regional road | Presence of the paved trail | No. of street intersections | No./presence of subway/rail stations | Length of subway/rail | No. of bus stops | No. of bus lines | No. of stations (available bikes) | Capacity of docks | Distance to nearby bike stations | Street tree/shadow | Street light/lamp | Station distance to CBD | Transfer distance to transit | Population/household density | Employment/job density |
| Dockless bikeshare | Feeder use for metro | Ni & Chen, 2020 (48) | | + | + |  |  |  |  |  |  |  | - |  |  |  |  | + |  |  | - | - |  | - | - |  |  |  |  |  |  |  |  |  |
|  |  | Liu et al., 2020 (58) | |  |  |  |  |  |  |  |  |  |  |  |  | + |  |  |  |  |  |  |  |  |  |  |  |  |  |  | - |  |  |  |
|  |  | Guo & He, 2020 (14) | |  |  | + |  |  | + |  |  |  | + |  |  | + |  |  |  |  | - | - |  | + |  |  |  |  |  |  | - | - |  |  |
|  |  | Guo et al., 2021 (21) | |  | - |  |  |  | + |  |  |  |  |  |  | + |  |  |  |  |  |  |  |  |  | + |  |  |  |  |  | - |  |  |
|  | General use | Shen et al., 2018 (50) | |  |  | - | + |  | + |  |  |  |  |  |  | + |  |  |  |  | + |  |  | + |  | + |  |  |  |  | + | - |  |  |
|  |  | Tu et al., 2019 (51) | | + |  | + |  |  | + |  |  |  | + |  |  |  | + | + |  |  | - |  |  | + |  |  |  |  |  |  |  |  |  |  |
|  |  | Chen et al., 2020 (28) | |  |  |  |  |  |  |  |  |  |  | - |  |  |  | + |  |  |  |  |  | + |  |  |  |  |  |  |  | - |  |  |
|  |  | Ji et al., 2020 (34) | | + | + |  |  |  |  |  |  |  |  |  |  |  |  |  |  |  |  | + |  |  |  |  |  |  |  |  | + |  |  |  |
|  |  | Lin et al., 2020 (61) | | + | NL |  |  |  | + |  | - | NL |  |  |  | + |  |  |  |  |  |  |  |  |  |  |  |  |  |  |  |  |  |  |
|  |  | Chen & Ye, 2021 (53) | |  |  | NL |  |  |  |  |  | NL |  | NL |  |  |  |  |  |  | NL | NL |  | NL | NL |  |  |  |  |  |  |  | NL | NL |
|  |  | Gehrke et al., 2021 (66) | |  |  |  |  |  |  |  |  |  |  |  |  |  |  |  |  |  |  |  |  |  |  |  |  |  |  |  |  |  | + | + |
| Docked bikeshare | Feeder use for metro | Ji et al., 2017 (72) | |  |  |  |  |  |  |  |  |  |  |  |  |  |  |  |  |  |  |  |  |  |  |  |  |  |  |  |  |  |  |  |
|  |  | Zhao & Li, 2017 (44) | |  |  |  |  |  |  | - |  |  | + |  |  |  |  | + | - |  |  |  |  |  | - | + |  |  |  |  | - |  |  |  |
|  |  | Lin et al., 2018 (35) | |  |  |  |  |  |  |  |  |  |  |  |  |  | - |  |  |  | - |  |  |  |  |  |  |  |  |  |  | + |  | + |
|  |  | Ji et al., 2018 (19) | |  |  |  | + |  |  |  |  | + |  |  |  |  |  |  |  |  |  | - |  | - |  | + | + |  |  |  |  | - | + |  |
|  |  | Liu et al., 2020 (59) | |  |  |  |  |  |  |  |  |  |  |  |  |  |  |  |  |  |  |  |  | - |  | - | + |  |  |  | - |  |  |  |
|  | General use | Buck & Buehler, 2012 (79) | |  |  |  |  |  |  |  |  |  |  |  |  | + |  |  |  |  |  |  |  |  |  |  |  |  |  |  |  |  | + |  |
|  |  | Rixey, 2013 (46) | |  |  |  |  |  |  |  |  |  |  |  |  | + |  |  |  |  |  |  |  |  |  | + |  |  |  |  |  |  | + | + |
|  |  | Mateo-Babiano et al., 2016 (54) | |  |  |  |  |  |  |  |  |  |  |  |  |  |  |  |  |  |  |  |  |  |  |  |  |  |  |  |  |  |  |  |
|  |  | Wang et al., 2016 (16) | |  |  |  |  |  |  |  | + |  |  | + |  |  |  |  |  | + |  |  |  |  |  |  |  | + |  |  | - |  |  | + |
|  |  | de Chardon et al., 2017 (80) | |  |  |  |  |  |  |  |  |  |  |  |  | + |  |  |  |  |  |  |  |  |  | + |  |  |  |  |  |  | + |  |
|  |  | Mattson & Godavarthy, 2017 (81) | |  |  |  |  |  |  |  | + |  |  |  |  |  |  |  |  |  |  |  |  |  |  |  | + |  |  |  |  |  | + |  |
|  |  | Duran-Rodas et al., 2019 (60) | | + |  |  |  |  |  |  |  |  | + | + |  |  |  |  |  |  |  | + |  | + |  |  |  |  |  |  |  |  |  |  |
|  |  | Wu et al., 2019 (82) | |  | + |  | + |  |  |  | + |  | - |  |  |  |  |  |  |  |  | + |  |  |  |  |  |  |  |  |  |  | + |  |
|  |  | Ji et al., 2020 (34) | | + | + |  |  |  |  |  |  |  |  |  |  |  |  |  |  |  |  | + |  |  |  |  |  |  |  |  |  |  |  |  |
|  |  | Zhao et al., 2021 (63) | *Arrival* | + |  |  |  |  |  | - | + | + | + | + |  | + |  |  |  |  |  |  |  | + |  |  | + | - |  |  |  |  |  | + |
|  |  |  | *Departure* | + |  |  |  |  |  | - | + | + | + | + |  | + |  |  |  |  |  |  |  | + |  |  | + | - |  |  |  |  |  | + |
|  |  | Lee & Noland, 2021 (65) | *Departure* | + |  |  |  |  |  |  |  |  |  |  |  |  |  |  |  |  |  | + |  |  |  |  |  |  |  |  |  |  |  | + |
|  |  | Wang & Chen, 2020 (62) | *Arrival* |  |  |  |  |  |  |  | + |  |  | + |  | + |  |  |  |  |  |  |  | + |  |  | + | - |  |  |  |  |  |  |
|  |  | Yang et al., 2020 (49) | *Arrival* |  |  |  |  |  |  |  |  |  |  |  |  |  |  |  |  |  |  |  |  |  |  |  | + |  |  |  | - |  |  | + |
|  |  |  | *Departure* |  |  |  |  |  |  |  |  |  |  |  |  |  |  |  |  |  |  |  |  |  |  |  | + |  |  |  |  |  |  |  |
|  |  | Tran et al., 2014 (37) | *Arrival* |  |  |  |  |  |  |  |  |  |  |  |  |  |  |  |  |  |  | + |  |  |  | + | + |  |  |  |  |  |  | + |
|  |  |  | *Departure* |  |  |  |  |  |  |  |  |  |  |  |  |  |  |  |  |  |  | + |  |  |  | + | + |  |  |  |  |  |  | + |
|  |  | Faghih-imani et al., 2017 (45) | *Arrival* |  |  |  |  |  |  |  |  | + |  | + |  |  |  |  |  |  |  |  |  |  |  | + | + |  |  |  |  |  | + |  |
|  |  |  | *Departure* |  |  |  |  |  |  |  |  | + |  | + |  |  |  |  |  |  |  |  |  |  |  | + | + |  |  |  |  |  | + |  |
|  |  | Faghih-imani et al., 2017 (18, 45) | *Arrival* |  |  |  |  |  |  |  |  | + |  | + | + |  |  |  |  |  |  |  |  |  |  | - | + |  |  |  |  |  | + |  |
|  |  |  | *Departure* |  |  |  |  |  |  |  |  | + |  | + | + |  |  |  |  |  |  |  |  |  |  |  | + |  |  |  |  |  | + |  |
|  |  | Kim et al., 2012 (83) | *Arrival* | + |  |  | + |  |  |  | + |  | + |  |  |  |  |  |  |  |  | + |  |  |  |  |  |  |  |  |  |  |  |  |
|  |  |  | *Departure* | + |  |  | + |  |  |  | + |  | + |  |  |  |  |  |  |  |  | + |  |  |  |  |  |  |  |  |  |  |  |  |
|  |  | Faghih-imani et al., 2014 (15) | *Arrival* |  |  |  |  |  |  |  |  |  |  | + |  | + | - | + |  |  |  | + |  |  |  | + |  |  |  |  | - |  |  | + |
|  |  |  | *Departure* |  |  |  |  |  |  |  |  |  |  | + |  | + | - | + |  |  |  | + |  |  |  | + |  |  |  |  | - |  |  | + |
|  |  | Faghih-imani & Eluru, 2016 (11) | *Arrival* |  |  |  |  |  |  |  |  |  |  | + |  | + |  |  | - |  |  | + |  |  |  |  |  |  |  |  | - |  |  |  |
|  |  |  | *Departure* |  |  |  |  |  |  |  |  |  |  | + |  | + |  |  | - |  |  | + |  |  |  |  |  |  |  |  | - |  |  |  |
|  |  | Faghih-imani & Eluru, 2016 (12) | *Arrival* |  |  |  |  |  |  |  |  |  |  | + |  |  |  |  |  |  |  | + | - |  |  |  |  |  |  |  |  |  | + |  |
|  |  |  | *Departure* |  |  |  |  |  |  |  |  |  |  | + |  |  |  |  |  |  |  | + | - |  |  |  |  |  |  |  |  |  | + |  |
|  |  | Faghih-imani et al., 2017 (18) | *Arrival* |  |  |  |  | - |  |  |  |  |  | + |  | + |  |  |  |  |  | + |  |  |  |  |  |  |  |  |  |  | + | + |
|  |  |  | *Departure* |  |  |  |  | - |  |  |  |  |  | + |  | + |  |  |  |  |  | + | - |  |  |  |  |  |  |  |  |  | + | + |
|  |  | El-Assi et al., 2017 (17) | *Arrival* |  |  |  |  |  |  |  | + |  |  |  |  |  |  |  |  |  |  | + |  |  |  | + | + |  |  |  |  |  | + | + |
|  |  |  | *Departure* |  |  |  |  |  |  |  | + |  |  |  |  |  |  |  |  |  |  | + |  |  |  | + | + |  |  |  |  |  | + | + |
|  |  | Sun et al., 2018 (43) | *Arrival* |  |  |  | - |  |  |  | - |  |  |  |  |  |  |  |  |  |  |  |  |  | + |  |  |  |  | + |  |  |  | + |
|  |  |  | *Departure* |  | - |  |  | - |  |  | - |  |  |  |  |  |  |  |  |  |  |  |  |  | + |  |  |  |  | + |  |  |  | + |
|  |  | Liu & Lin, 2019 (20) | *Arrival* |  |  |  | + |  | + |  |  | + |  |  |  |  |  |  |  |  |  |  |  |  |  |  | + |  |  |  |  | - |  | - |
|  |  |  | *Departure* |  |  |  | + |  |  |  |  |  |  |  |  |  | + |  |  |  |  |  |  |  |  | + | - |  | + | + | - | - | - | + |
|  |  | Noland et al., 2016 (69) | *Weekday* |  |  |  |  |  |  |  |  |  |  |  |  |  |  |  |  |  |  |  |  |  |  | + |  |  |  |  |  |  | + | + |
|  |  |  | *Weekend* | + |  |  |  |  | + |  |  |  |  |  |  | + |  |  |  |  |  |  |  |  |  | + |  |  |  |  |  |  | + |  |
|  |  | Faghih-imani & Eluru, 2016 (12) | *Weekday* |  |  |  |  |  |  |  |  |  |  |  |  |  |  |  |  |  |  |  |  |  |  |  |  |  |  |  |  |  |  |  |
|  |  |  | *Weekend* |  |  |  |  | + |  |  |  |  |  |  |  |  |  |  |  |  |  |  |  |  |  |  |  |  |  |  |  |  |  |  |
|  |  | El-Assi et al., 2017 (17) | *Weekday* |  |  |  |  |  |  |  | + |  |  |  |  |  |  |  |  |  |  | + |  |  |  | + | + |  |  |  |  |  | + | + |
|  |  |  | *Weekend* |  |  |  |  |  |  |  |  |  |  |  |  |  |  |  |  |  |  |  |  |  |  |  | + |  |  |  |  |  | + |  |
|  |  | Faghih-imani et al., 2017 (18) | *Weekday* |  |  |  |  |  |  |  |  |  |  |  |  |  |  |  |  |  |  |  |  |  |  |  |  |  |  |  |  |  |  |  |
|  |  |  | *Weekend* |  |  |  |  | + |  |  |  |  |  |  |  |  |  |  |  |  |  |  |  |  |  |  |  |  |  |  |  |  |  |  |
|  |  | Zhang et al., 2017 (13) | *Weekday* |  |  |  |  |  | + |  |  |  |  |  |  | + |  | + |  |  |  |  |  |  |  | - |  |  |  |  | - |  | + |  |
|  |  |  | *Weekend* |  |  |  |  |  | + |  |  |  |  |  |  | + |  | + |  |  |  |  |  |  |  | - | + |  |  |  | - |  | + |  |
|  |  | Alcorn & Jiao, 2019 (84) | *Weekday* |  |  |  |  |  |  |  |  |  |  |  |  | + | + |  |  |  |  |  |  |  |  |  |  |  |  |  |  |  |  |  |
|  |  |  | *Weekend* |  |  |  |  |  |  |  |  |  |  |  |  | + |  |  |  |  |  |  |  |  |  |  |  |  |  |  |  |  |  |  |
|  |  | Lin et al., 2020 (47) | *Weekday* | + | + |  | + |  |  |  |  |  | + |  |  | + |  |  |  |  |  | + |  | + |  |  |  |  |  |  |  |  |  |  |
|  |  |  | *Weekend* | + | - |  | + |  |  |  |  |  | + |  |  | + |  |  |  |  |  | + |  | + |  |  |  |  |  |  |  |  |  |  |

Note: **+** means a positive effect. - means a negative effect. NL means a nonlinear relationship.
